# Psychometric Network Characteristics of Anxiety and Depression Among Chinese Physical Education Teachers

**DOI:** 10.3390/bs16091534

**Published:** 2026-08-31

**Authors:** Mei Wu, Chengkui Yao

**Affiliations:** 1 School of Physical Education, Guizhou University of Finance and Economics, Guiyang 550025, China; wumei@mail.gufe.edu.cn; 2 School of Psychology, Guizhou Normal University, Guiyang 550025, China

**Keywords:** anxiety, depression, symptomatic characteristics, psychometric network, physical education teachers

## Abstract

The symptomatic characteristics of anxiety and depression among physical education (PE) teachers have not received sufficient attention in existing research. However, these characteristics are crucial for formulating targeted interventions for such issues. This study aimed to explore the symptomatic characteristics of the comorbid anxiety–depression network among Chinese PE teachers, so as to initially provide a direction for targeted interventions. A total of 519 Chinese PE teachers (69.56% male; *M*_age_ = 31.58, *SD*_age_ = 8.18) were recruited as research subjects, who were asked to complete a set of questionnaires covering demographic information, anxiety scales and depression scales. Both psychometric network analysis and Network Comparison Tests (NCTs) were adopted to identify the core symptoms, bridge symptoms, overlapping items, and gender-specific effects in the anxiety–depression comorbidity network. The results indicated that the psychometric network structure was relatively stable. “Psychomotor change” was identified both as the core EI (1.26) and bEI (0.62) indicators, while “depressed mood” also served as a key bEI (0.70) indicator. Moreover, “restlessness”, “irritability”, “self-deprecation”, and “psychomotor change” were the four overlapping symptoms. Additionally, network comparison yielded mixed findings across gender groups: symptom interconnections patterns were largely comparable, while global strength differed significantly between male and female PE teachers. Collectively, symptom nodes demonstrated heterogeneous importance within the network. Nodes occupying core positions may play a crucial role in guiding prevention and intervention for anxiety and depressive symptoms.

## 1. Introduction

As a core force in school education, PE teachers have long been shouldered multiple responsibilities and endured long-term pressures from all walks of their work, which render them a high-risk group for anxiety and depression (Agyapong et al., 2022; X. Li et al., 2025; Ma et al., 2025; Xu, 2019). Such mental health status quo may directly exert an impact on the quality of physical education and the effectiveness of fostering students’ physical health (Agyapong et al., 2022; X. Li et al., 2025). Existing research is mostly limited to prevalence statistics and factor/outcome exploration of their anxiety and depression status (Agyapong et al., 2022; X. Li et al., 2025; Lu & Zhang, 2018; von Haaren-Mack et al., 2020), and traditional latent variable models cannot reveal inter-symptom relationships to support precise interventions (Luo et al., 2024). Thus, this study adopted network analysis as the research method, took Chinese PE teachers as the research subjects, and collected symptom-related data via questionnaires. On this basis, an anxiety–depression symptom network model was constructed to identify core symptoms, which is expected to provide theoretical support and practical reference for formulating targeted mental health intervention strategies for Chinese PE teachers.

With the advancement and implementation of educational reform policies, the status of physical education (PE) has been gradually elevated, while the professional pressure on Chinese PE teachers has exhibited features of diversification and superimposition. They face not only long-standing traditional predicaments, such as heavy safety responsibilities (X. Li et al., 2025), disciplinary bias and undervaluation-related pressure (Zhu & Li, 2019), a “glass ceiling” in career development (Zhu & Li, 2019), overwork, and health crisis (L. He et al., 2025; X. Li et al., 2025; Lu & Zhang, 2018), but also new challenges brought by educational reform initiatives. For example, the “double reduction” policy has integrated PE into after-school services, thereby increasing PE teachers’ workload (L. He et al., 2025; J. Li et al., 2024b). Although policies explicitly stipulate that PE teachers shall enjoy equal treatment with teachers of other subject in professional title evaluation and recognition, the transformation of the actual evaluation mechanism lags behind. Some schools still adopt evaluation methods applicable to cultural courses, ignoring the practical and comprehensive characteristics of PE teaching, which has inadvertently increased PE teachers’ assessment pressure and work burden (C. He, 2025; Zhu & Li, 2019). In addition, driven by policy orientation and teaching practice, PE teachers are in urgent need of professional capability upgrading, as the pace and difficulty of their professional updating have been significantly accelerated. Especially in the digital age, PE teachers are also required to enhance their professional skills by learning and mastering high-tech technologies (Ma et al., 2025).

The aforementioned challenges have severely undermined PE teachers’ physical and mental well-being, leading to poor physical health and reduced occupational mental health, including increased anxiety and depression (Agyapong et al., 2022; L. He et al., 2025). Internationally, the physical and psychological toll on PE teachers is well documented. An empirical study from Québec reported high incidence of physical injuries—such as lower back pain—among school PE teachers, with a 55% annual recent lesion rate per teacher (65% for females and 51% for males; Lemoyne et al., 2007). A systematic review further indicated that 20–25% of PE teachers experience high burnout levels as a consequence of chronic work-related pressures (von Haaren-Mack et al., 2020). In China, the mental health profile of the PE teachers is similarly concerning, characterized by both high detection rates and uneven symptom distributions across dimensions. Studies across different regions and educational levels consistently reveal elevated psychological burden: A SCL-90 survey of 207 university PE teachers in Jiangsu Province found exceptionally high positive rates across all factors, with depression reaching 97.56% and anxiety 87.80% (Q. Liu et al., 2006), and a separate study of university PE teachers in Wuhan reported an 11.9% comorbid detection rate for anxiety and depression (Leng & Kong, 2014). At the secondary school level, a study of 500 middle school PE teachers in Shaanxi Province reported that 73.86% exhibited varying degrees of psychological distress, substantially exceeding the average level among general teacher groups (Zuo, 2009). Among primary and secondary school teachers in Jiangsu Province (including a PE teacher subsample), detection rates for somatization, depression, and anxiety were 19.14%, 12.89%, and 10.16%, respectively (A. Liu & Liu, 2015). A large-scale systematic analysis of Chinese teachers across all educational levels (preschool through university) since 2000—encompassing over 570,000 participants—reported national pooled prevalence rates of 16.1% for anxiety and 18.8% for depression among the general teaching population (Jin & Yu, 2025). Against this national baseline, the elevated rates observed in PE teacher samples underscore the heightened vulnerability of this occupational subgroup. Notably, female PE teachers face disproportionately greater mental health challenges. Despite comparable compensation and benefits, female PE teachers endure unprecedented mental stress stemming from gender discrimination, professional title promotion, and child education (Lu, 2018). Collectively, these findings reveal a substantial psychological burden beneath the “health guardian” occupational stereotype of PE teachers, and this phenomenon remains prevalent and severe across the teaching profession (Agyapong et al., 2022; X. Li et al., 2025).

Building on prior research, anxiety and depression are recognized as the most prevalent mental disorders and they exhibit the highest comorbidity rate among all mental illness (Cai et al., 2024; Luo et al., 2024; Morneau-Vaillancourt et al., 2020). As demonstrated by most empirical studies, the co-occurrence rate of anxiety and depression ranges roughly from 30% to 78% (Choi et al., 2020; Gaspersz et al., 2018). With regard to the long-term challenges encountered by PE teachers, the combination of the two is more severe than either alone, posing a higher risk of suicide, stronger resistance to treatment, and greater destructive power (Tiller, 2012); resulting in more physical, psychological, social, and work-related impacts, such as weight fluctuation (de Wit et al., 2015), impaired immune function (Wu et al., 2021), chronic heart disease (Scherrer et al., 2010), and impaired emotional well-being (Kayaalp et al., 2020). As a result, these conditions not only lower PE teachers’ life satisfaction, but also impose economic burden on families and society, posing substantial challenges to public health issues (P. Zhang et al., 2023). Psychological researchers have consistently emphasized the importance of PE teachers’ mental health in ensuring instructional quality and promoting student development (Agyapong et al., 2022; X. Li et al., 2025). However, most existing mental health research—with a focus on issues such as anxiety and depression—centers on groups including chronic diabetes (Basiri et al., 2023), elder people with disabilities (P. Zhang et al., 2023), pregnant woman (Ou et al., 2025), college students (J. Li et al., 2024a; Su & Liu, 2025), general teachers (Agyapong et al., 2022; Carroll et al., 2022) and other special populations, whereas systematic research specifically targeting PE teachers remains relatively scarce.

Numerous studies have generated substantial thoughtful and comprehensive insights into anxiety and depression from the traditional lens of latent variables. Specifically, these studies characterize participants’ anxiety and depression levels using the total scores of anxiety and depression scales, and examine the antecedent and outcome variables associated with anxiety and depression through an assessment of their correlations with other variables, such as burnout (Amirhosseini et al., 2021), physical activities (Bogaert et al., 2014), and motivational climate (Melguizo-Ibáñez et al., 2025). However, many studies have indicated that the total score approach may not be feasible when approaching anxiety and depression issues (Luo et al., 2024), and this is mainly due to the following two reasons: firstly, the total score approach merely captures the shared variance across all symptoms, while overlooking information pertinent to the intraindividual evolution of psychopathology (Bringmann et al., 2015; Fried & Nesse, 2015); secondly, these two disorders not only overlap and frequent co-occurrence in symptomatology (Zbozinek et al., 2012) but also exhibit potential genetic overlaps identified in genome-wide association study (Morneau-Vaillancourt et al., 2020). In other words, anxiety and depression are not mutually exclusive disease categories, challenging the traditional categorical view of these conditions as distinct entities (Harlev et al., 2025; Vink et al., 2008). As a viable alternative, dimensional models hold greater promise for charactering the complex, overlapping architecture of psychopathological symptoms.

Within this dimensional framework, network analysis stands out as a robust analytical tool for dissecting the complex interrelationships among symptoms (Harlev et al., 2025; Park & Kim, 2020). Distinct from traditional diagnostic approaches, this method does not presuppose that the symptoms are affiliated with a specific disease category; instead, it incorporates all symptoms into the same continuous relational network, where symptoms (i.e., the items that constitute the scale) are regarded as nodes and the connections between them are defined as the edges. Specifically, in the symptom network, the size of each node reflects the coreness of the corresponding symptom (for instance, in the anxiety–depression network, “depressed mood” and “excessive worry” are likely to be core nodes (Bian et al., 2024; J. Li et al., 2023; Peng et al., 2022); the distribution of edges illustrates the direct or indirect association paths among symptoms (e.g., the chain association of “nervousness → sleep disturbance → fatigue → depressed mood”); the overall layout of the network demonstrates that anxiety and depression symptoms do not form completely isolated clusters, but rather a continuous spectrum with mutual infiltration, which perfectly echoes the advocacy of “non-categorization and continuity” in the dimensional model (Vink et al., 2008). Therefore, symptom-based network analysis plays a pivotal role in the research on anxiety–depression comorbidity (Luo et al., 2024).

To date, numerous empirical studies have employed network analysis to explore the associations between anxiety and depressive symptoms in various populations including nurses (Peng et al., 2022), psychiatric sample (Beard et al., 2016; Kaiser et al., 2021), individual with diabetes (Y. Zhang et al., 2024), older adults (Bian et al., 2024), and college students (J. Li et al., 2024a; Luo et al., 2024). This line of research has yielded a wealth of meaningful findings. Specifically, the majority of the studies have found that anxiety symptoms—such as “uncontrollable worry”, “excessive worry” and “difficulty relaxing”—are more central than depressive symptoms (Harlev et al., 2025; Luo et al., 2024; Shen & Wang, 2023), while “depressed mood” is a frequently identified as a core and bridging symptom among depressive symptoms (Bian et al., 2024; Peng et al., 2022). Furthermore, all existing studies have demonstrated that certain key nodes, acting as bridge symptoms, connect anxiety and depressive symptoms, and it is widely believed that these bridge symptoms play an instrumental role in the development and maintenance of the comorbidity between the two disorders (Ren et al., 2021). Additionally, these network structures have been proved to be stable across genders and over time (Bian et al., 2024; J. Li et al., 2024a). Despite these notable findings, some uncertainties remain. First, most studies have suggested that symptom overlap between anxiety and depressive symptoms may underlie the comorbidity of these two disorders (Boschloo, 2018; Ren et al., 2021; Zbozinek et al., 2012). However, there are merely limited studies that have utilized community testing to identify specific overlapping symptoms (Harlev et al., 2025; Kaiser et al., 2021). This inadequacy of available evidence regarding the overlap between anxiety and depressive symptoms is unfavorable for intervention research on their comorbidity. Second, the characteristics of anxiety and depressive symptoms among PE teachers remain undetermined, as no studies have employed network analysis to examine anxiety and depressive symptoms in this population. Considering the high levels of pressure and severe mental health challenges faced by Chinese PE teachers, it may not be appropriate to generalize the aforementioned findings to this specific group. Psychological researchers have noted that providing information on symptoms, diagnosis, and evidence-based treatment constitutes the first step in assisting patients with anxiety and depression (Szuhany & Simon, 2022). Therefore, it is imperative to employ symptom-based network analysis to model and analyze the comorbidity of anxiety and depressive symptoms in PE teachers.

This study represents the first attempt to address this gap by employing network analysis to model the complex interactive relationship between anxiety and depressive symptoms in Chinese PE teachers. The primary objectives were to identify central and bridging symptoms, detect the community structure within the symptom network, and examine the gender difference in network characteristics, thereby providing a theoretical basis for future interventions. We hypothesized that (a) symptoms such as “uncontrollable worry”, “excessive worry” and “difficulty relaxing” would emerge as the most central symptoms, while “depressed mood” would function as the bridging symptom within the network; (b) the overall network structure would remain stale across the whole sample and exhibit invariance across genders; and (c) a certain degree of symptom overlap would be observed in the anxiety–depression symptom network.

## 2. Materials and Methods

### 2.1. Participants

A total of 522 full-time PE teachers were initially recruited from primary and secondary schools across multiple regions in Guizhou Province, China (e.g., Guiyang [41.25%], Zunyi [18.34%], Qian Dongnan [20.11%], and Liu Panshui [20.30%]), with data collected through an online self-administered questionnaire. To ensure data integrity and analytical rigor, we conducted comprehensive outlier screening: three cases were excluded due to meeting both univariate outlier criteria (|*Z*| > 3) and multivariate outlier criteria (Mahalanobis distance, *p* < 0.001), with over 50% of their data points classified as outliers. This screening process yielded a final valid sample of 519 participants, all of whom met the study’s inclusion criteria: (a) current full-time employment as a PE teacher; (b) voluntary, informed consent to participate; (c) ability to complete the questionnaire independently. Exclusion criteria included incomplete responses, systematic response bias, and outlier status as defined above.

The final sample comprised 361 male (69.56%) and 158 female (30.44%) participants, with ages ranging from 20 to 57 years (*M* = 31.58, *SD* = 8.18). Age stratification revealed that 305 participants (61.61%) were below 35 years old, 98 (19.79%) were aged 35–40 years old, and 92 (18.59) were 40 years or older; 24 participants (4.62%) had missing data. Regarding educational attainment, the majority held a bachelor’s degree (*n* = 420, 80.92%), followed by master’s degree holders (*n* = 53, 10.21%), associate degree holders (*n* = 39, 7.51%), doctorate holders (*n* = 4, 0.77%), and high school graduates (*n* = 3, 0.59%). In terms of teaching stage, 352 participants (67.82%) taught at the junior high school level or above, while 167 (32.18%) taught in primary schools. This demographically diverse sample ensures representativeness of PE teachers across age groups, educational backgrounds, and teaching stages, providing a robust foundation for investigating mental health in this population.

### 2.2. Procedure

This study adopted an online questionnaire-based cross-sectional design, and data were collected through a professional online survey platform (https://www.wjx.cn/). Prior to participating in the survey, all potential participants were presented with an electronic informed consent form, which clearly elaborated on the study’s purpose, research procedures, estimated completion time (approximately 15–20 min), potential risks, and the academic use of collected data. Participation was entirely voluntary, and participants had the right to withdraw from the survey at any time without any penalty or adverse impact, with no questions asked. Only after participants read and electronically signed the informed consent form could they access the formal questionnaire. All responses were collected anonymously; no personally identifiable information (e.g., name, contact information, IP address) was recorded to protect participants’ privacy. The questionnaire was designed with logical consistency, and participants were instructed to answer truthfully and independently. After data collection, all raw data were encrypted and stored in a password-protected electronic database, with strict confidentiality maintained in line with academic ethics and relevant regulations. The present study was approved by the Institutional Review Board of School of Psychology of Guizhou Normal University (GZNUPSY.N.202509E [0008]).

### 2.3. Measures

#### 2.3.1. Generalized Anxiety Disorder 7-Item Scale (GAD-7)

The GAD-7 is a brief, reliable self-administered instrument developed to screen for and evaluate the severity of anxiety disorder (Spitzer et al., 2006). Comprising seven items, the scale measures the frequency of core anxiety symptoms (e.g., nervousness, excessive worry, restlessness) experienced by participants over the past two weeks. Each item is rated on a 4-point Likert scale, ranging from 0 (“Not at all”) to 3 (“Nearly every day”), with a total score ranging from 0 to 21. An optimal cut-off score of 10 has been identified, with a sensitivity of 0.89 and specificity of 0.82 for identifying GAD (Spitzer et al., 2006). The Chinese version of GAD-7 has been used successfully in diverse Chinese contexts (Luo et al., 2024; Ren et al., 2021). The Cronbach’s α coefficient, composite reliability (CR), and the average variance extracted (AVE) for the GAD-7 in this study were 0.92, 0.92, and 0.62, respectively.

#### 2.3.2. 9-Item Patient Health Questionnaire (PHQ-9)

The PHQ-9 is a widely used self-report scale for screening and assessing depressive symptoms and potential depressive disorders (Kroenke et al., 2001). As a component of the larger Patient Health Questionnaire (PHQ), it can be used as a standalone instrument and assesses nine core symptoms of major depressive disorder (MDD) consistent with DSM-5 diagnostic criteria (e.g., anhedonia, depressed mood, suicidal ideation). Participants rate the frequency of each symptom over the past two weeks on a 4-point Likert scale (0 = “Not at all” to 3 = “Nearly every day”), with a total score ranging from 0 to 27. A cutoff of 7 demonstrates an equal sensitivity and specificity rate of 86% (W. Wang et al., 2014). Previous studies have demonstrated the high reliability and validity of the Chinese version of the PHQ-9 in screening for depression in various populations (Cai et al., 2024; Luo et al., 2024; X. Y. Wang et al., 2025). The Cronbach’s α coefficient, CR, and AVE for the PHQ-9 in this study were 0.92, 0.92, and 0.56, respectively.

### 2.4. Statistical Analysis

All descriptive statistical analyses were performed using IBM Statistical Package for the Social Sciences (SPSS, Version 26.0), while others, including scale reliability and network analysis, were conducted via the R statistical software (Version 4.5.2).

A cross-sectional Gaussian Graphical Model (GGM) was estimated with ℓ_1_ regularization (graphical lasso) based on polychoric correlation matrices. The regularization parameter was selected via Extended Bayesian Information Criterion (EBIC) with γ = 0.50 (Epskamp & Fried, 2018), and edge weights were regularized partial correlations.

Community detection was implemented via the Clique Percolation Method (CPM). This approach is particularly advantageous for identifying symptom overlap within psychometric networks, as it accommodates node membership in multiple communities simultaneously (Palla et al., 2005; Reichardt & Bornholdt, 2006). To derive a stable, interpretable, and structurally robust community structure, the optimal parameters—including the clique size (*k*, defining the minimum size of constituent *k*-cliques) and the intensity threshold (*i*, specifying the minimum connection strength required for node inclusion into a community)—were determined through a joint assessment of the largest component ratio, chi-square value, and entropy via permutation testing (Lange, 2022).

Two centrality indices were computed: expected influence (EI) and bridge expected influence (bEI). The EI index was adopted to characterize the connectivity of each node, instead of the “Strength” index, as previous research has demonstrated that the EI index exhibits superior performance in the presence of negative correlations (Robinaugh et al., 2016). bEI identifies symptoms that facilitate cross-community connections between anxiety and depression clusters, critical for interpreting comorbidity. Betweenness and closeness were not reported due to low stability in psychological symptom networks (Epskamp et al., 2018).

Stability of centrality indices was evaluated via case-drop bootstrap (1000 resamples) and CS coefficient (should not below 0.25 and preferably be above 0.50) (Epskamp et al., 2018). Edge accuracy was assessed using bootstrapped 95% confidence intervals.

Separate Gaussian Graphical Models for female and male subgroups were estimated following the identical estimation pipeline used for the full sample, employing the graphical lasso with EBIC model selection (default = “EBICglasso”). The Network Comparison Test (NCT) was applied to statistically compare the overall network structure between male and female subgroups (van Borkulo et al., 2023). The NCT examined differences in network invariance, global strength, edge weight, and centrality characteristics across gender-based networks. The permutation test was performed with 1000 permutations to ensure stable *p*-value estimation. We implemented alternative correlation estimators (Pearson correlations) as cross-checks to evaluate whether substantive findings are sensitive to the choice of correlation matrix in the accuracy examination process. Given the limited sample size in the female group (158) and the multiple testing of 120 edges and 32 centrality indices, the False Discovery Rate (FDR) procedure was applied to adjust all *p*-values to control for Type I error inflation, with a significance level set at α = 0.05.

All network analyses were implemented using R statistical software (Version 4.5.2), with packages of qgraph, bootnet, igraph, EGAnet, Mgm, CliquePercolation, and NetworkComparisonTest.

## 3. Results

### 3.1. Descriptive Information of the Study Sample

**Figure 1:**
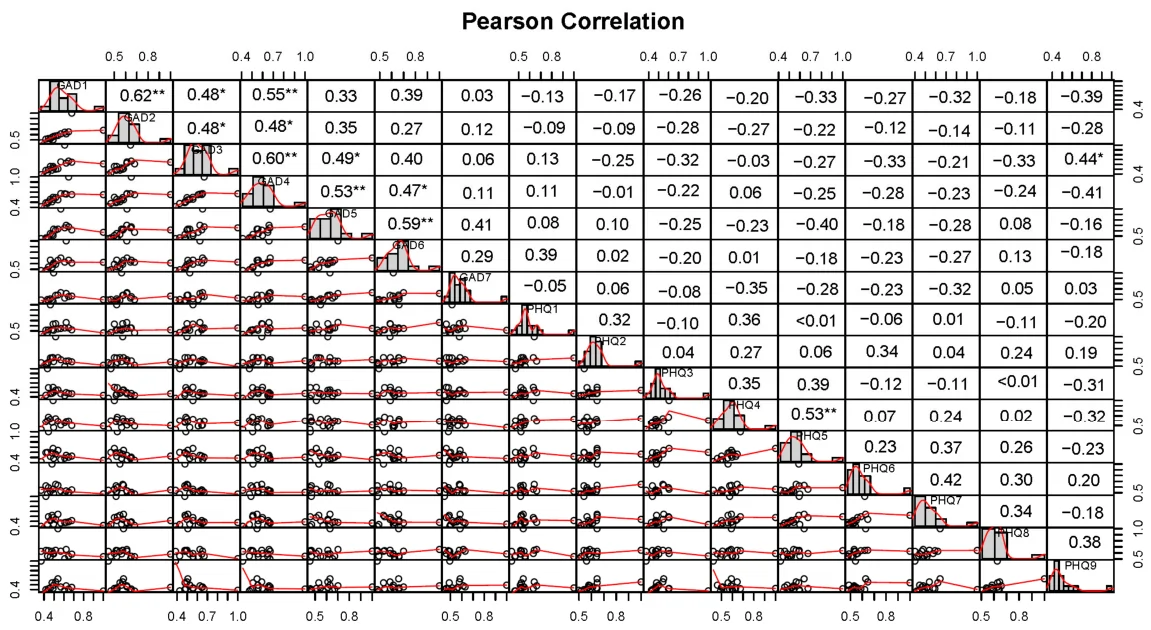
Bivariate correlation matrix of GAD-7 and PHQ-9 items. Diagonal panels display the histogram and density curve (red-colored line) for each item-score distribution. The upper-triangle matrix presents pairwise Pearson correlation coefficients of GAD-7 and PHQ-9 items with asterisks indicating significance (** *p* < 0.01, * *p* < 0.05). The lower-triangle panels contain scatterplots fitted with linear regression lines (red-colored line) to visualize the bivariate linear association between variables.

A total of 519 Chinese PE teachers’ data were analyzed. The mean total score for the GAD-7 scale and PHQ-9 scale were 4.19 (standard deviation [*SD*] = 4.59) and 4.45 (*SD* = 5.15), respectively. Furthermore, no significant gender differences were observed for total GAD-7 scores (*M*_male_ = 4.16, *SD* = 4.76; *M*_female_ = 4.25 *SD* = 4.18; *t* = −0.22, *p* = 0.823, Cohen’s *d* = −0.02) or total PHQ-9 scores (*M*_male_ = 4.29, *SD* = 5.20; *M*_female_ = 4.81, *SD* = 5.03; *t* = −1.07, *p* = 0.286, Cohen’s *d* = −0.10). Regarding anxiety, more male PE teachers (*n* = 49, *M* = 13.73, *SD* = 2.68) scored above the cutoff of 10 compared with their female counterparts (*n* = 19, *M* = 12.47, *SD* = 2.50). A consistent pattern emerged in the depression data: 101 (*M* = 11.30, *SD* = 4.34) males and 55 (*M* = 10.56, *SD* = 3.77) females scored above the cutoff of 7. The means, *SD*s, predictability of all the GAD-7 and PHQ-9 items, as well as additional gender-specific information, are presented in Table 1 and Table 2. The strongest bivariate correlation among anxiety symptoms was observed between GAD1 (“nervousness”) and GAD2 (“uncontrollable worry”, 0.62, *p* < 0.01), GAD3 (“excessive worry”) and GAD4 (“difficulty relaxing”, 0.60, *p* < 0.01) and GAD5 (“restlessness”) and GAD6 (“irritability”, 0.59, *p* < 0.01). Among depression symptoms, the strongest bivariate correlations were found between PHQ4 (“fatigue”) and PHQ5 (“appetite change”, 0.53, *p* < 0.01), PHQ6 (“self-deprecation”) and PHQ7 (“poor concentration”, 0.42), and PHQ8 (“psychomotor change”) and PHQ9 (“suicidal ideation”, 0.38). Between the two disorders, the strongest bivariate correlation was identified between GAD3 (“excessive worry”) and PHQ9 (“suicidal ideation”, 0.44, *p* < 0.05). The detailed bivariate correlation matrix of the GAD-7 and PHQ-9 items is shown in Figure 1.

### 3.2. Network Structure and Centrality Measure Analysis

**Figure 2:**
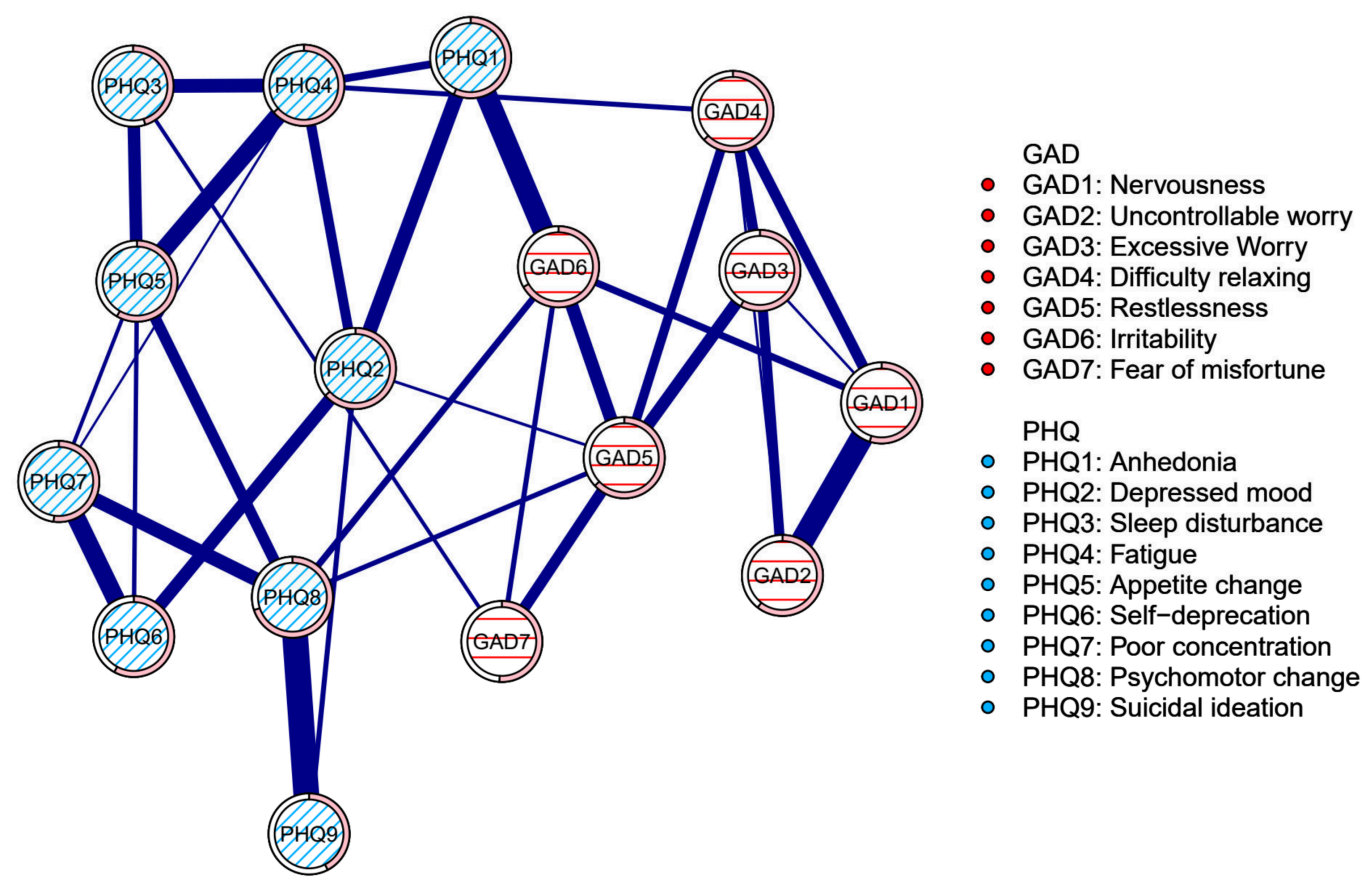
The psychometric structure of the anxiety–depression symptom network. Nodes (GAD-7 in red, PHQ-9 in blue) represent individual symptoms. Dark blue edges indicate positive, and red edges indicate negative associations; edge thickness reflects correlation strength. Weak associations were shrunk to zero via graphical lasso. The pink node ring indicates predictability (0 = no prediction, 1 = perfect prediction), measured by explained variance, representing the ability to predict one node by others in the network.

**Figure 3:**
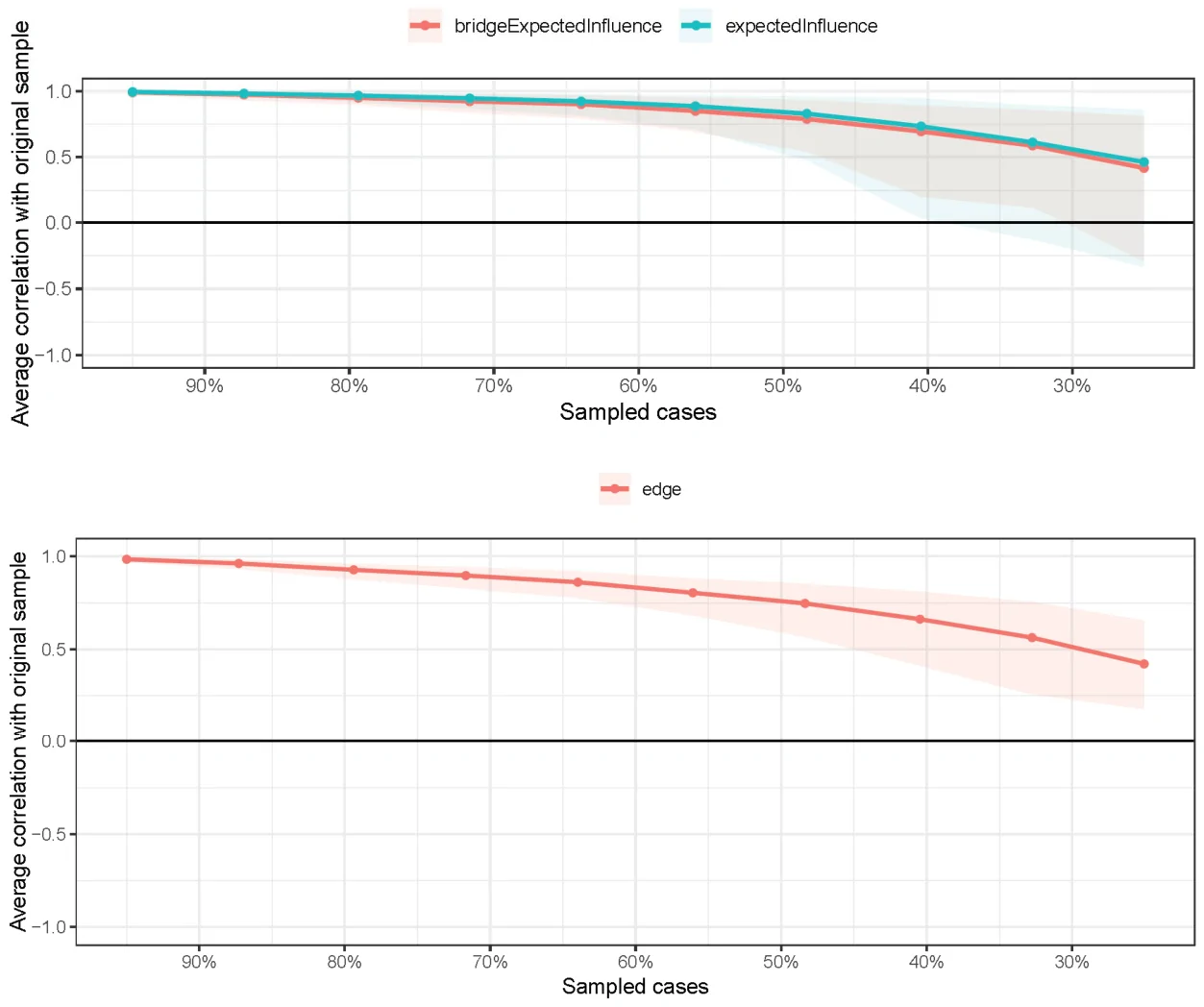
The correlation stability coefficients (CS-coefficient) for EI and bEI.

Figure 2 illustrates the network structure of the anxious depression symptom network. A total of 16 symptom nodes related to anxiety and depression formed 120 links, among which 97 were effective non-zero edges, accounting for 80.83% of the total links. The mean weight of the entire network was 0.06. From a visual standpoint, the internal links among anxiety symptoms or among depression symptoms were more closely knit and robust than the cross-links between anxiety and depression symptoms. The strongest associations within anxiety symptoms, within depression symptoms, and between these two disorders were as follows: GAD1 (“nervousness”) and GAD2 (“uncontrollable worry”) with a weight of 0.21; PHQ8 (“psychomotor change”) and PHQ9 (“suicidal ideation”) with a weight of 0.20; and GAD6 (“irritability”) and PHQ1 (“anhedonia”) with a weight of 0.17, respectively. The average predictability of the entire network was 0.59, among which GAD6 (“irritability”) in the anxiety symptom group and PHQ8 (“psychomotor change”) in the depression symptom group exhibited the highest predictability (see Table 1). The entire anxious depression network demonstrated good stability. Specifically, bootstrap 95% CI for edge weights indicated minimal estimation bias, as most sample edge weights lay within the confidence bands and aligned closely with bootstrap means. While confidence intervals for weaker edges overlapped zero, stronger positive edges exhibited stable non-zero associations (refer to Supplementary Materials Figure S1). Furthermore, compared with other edges, all the aforementioned strongest edges were statistically significant (see Supplementary Materials Figure S2). The CS-coefficient of the entire network edges was 0.52 (see Figure 3).

**Figure 4:**
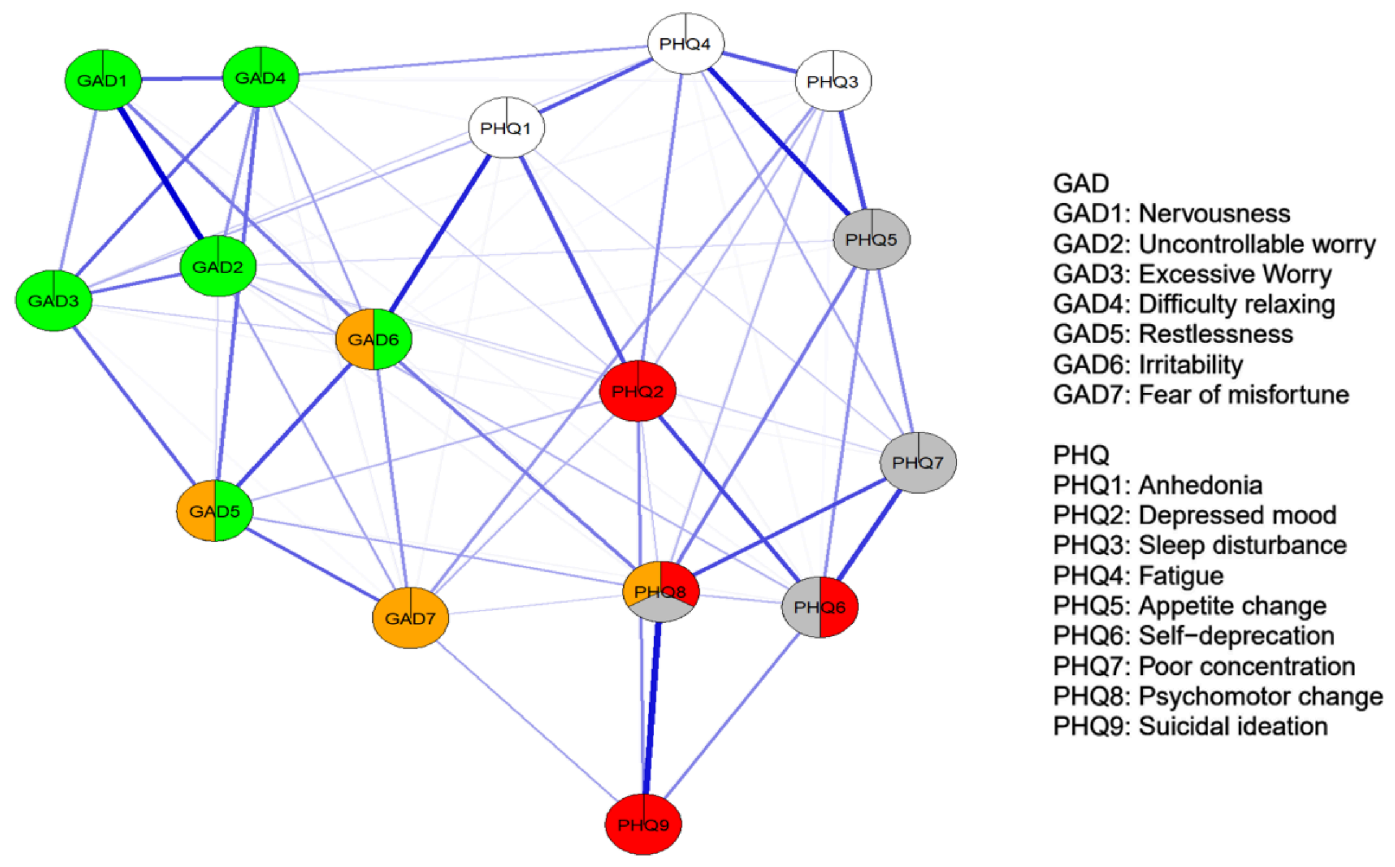
Result of the Community Percolation Analysis. Node colors correspond to four communities: red for Autoaggression Cluster, green for Psychomotor and Cognitive Disturbance Cluster, gray for Anxiety Arousal Cluster, and orange for Anxiety–Depression Comorbidity Cluster. GAD5, GAD6, PHQ6, and PHQ8 are shared nodes overlapping between related communities, while PHQ1, PHQ3, and PHQ4 are isolated nodes not included in any community due to weak connectivity.

A permutation test was conducted to determine the optimal parameter for the CPM. Results identified a clique size (*k*) of 4 and an intensity threshold (*i*) of 0.129 as the optimal values. Under these parameters, the corresponding entropy value fell within the 95% confidence interval [1.81, 1.95], confirming the stability and robustness of the community structure. CPM analysis identified four distinct communities. The first community comprised items from the PHQ, including PHQ2 (“depressed mood”), PHQ6 (“self-deprecation”), PHQ8 (“psychomotor change”), and PHQ9 (“suicidal ideation”). Since these items convey concepts related to negative self-evaluation and self-harm, this community was designed as Autoaggression Cluster. The second community included PHQ5 (“appetite change”), PHQ6 (“self-deprecation”), PHQ7 (“poor concentration”), and PHQ8 (“psychomotor change”). In consideration of its characteristic of integrating mental events and somatic behaviors, this community was defined as the Psychomotor and Cognitive Disturbance Cluster. The third community consisted of items from the GAD, namely GAD1 (“nervousness”), GAD2 (“uncontrollable worry”), GAD3 (“excessive worry”), GAD4 (“difficulty relaxing”), GAD5 (“restlessness”), and GAD6 (“irritability”). Given that these items comprehensively covered the full spectrum of anxiety arousal, this community was named the Anxiety Arousal Cluster. The last community included GAD5 (“restlessness”), GAD6 (“irritability”), GAD7 (“fear of misfortune”), and PHQ8 (“psychomotor change”). As this was the only community encompassing both anxiety- and depression-related symptoms, it was designated the Anxiety–Depression Comorbidity Cluster. Items like PHQ1 (“anhedonia”), PHQ3 (“sleep disturbance”), and PHQ4 (“fatigue”) were excluded in any community. Notably, this study identified GAD5, GAD6, PHQ6, and PHQ8 as four overlapping nodes between communities. Specifically, PHQ6 (“self-deprecation”) and PHQ8 (“psychomotor change”) connect the two depression-related communities (Autoaggression Cluster and Psychomotor and Cognitive Disturbance Cluster), while GAD5 (“restlessness”) and GAD6 (“irritability”) link the Anxiety Arousal Cluster and the Anxiety–Depression Comorbidity Cluster. This reflects the overlap of symptoms, which is consistent with the core advantage of the CPM that allows nodes to belong to multiple communities (refer to Figure 4). To test the identified community structure remained stable, we performed a sensitivity analysis after excluding these four overlapping items. The results further supported the four-community structure identified in the present study (for full details of this sensitivity analysis, see Supplementary Materials Table S1).

**Figure 5:**
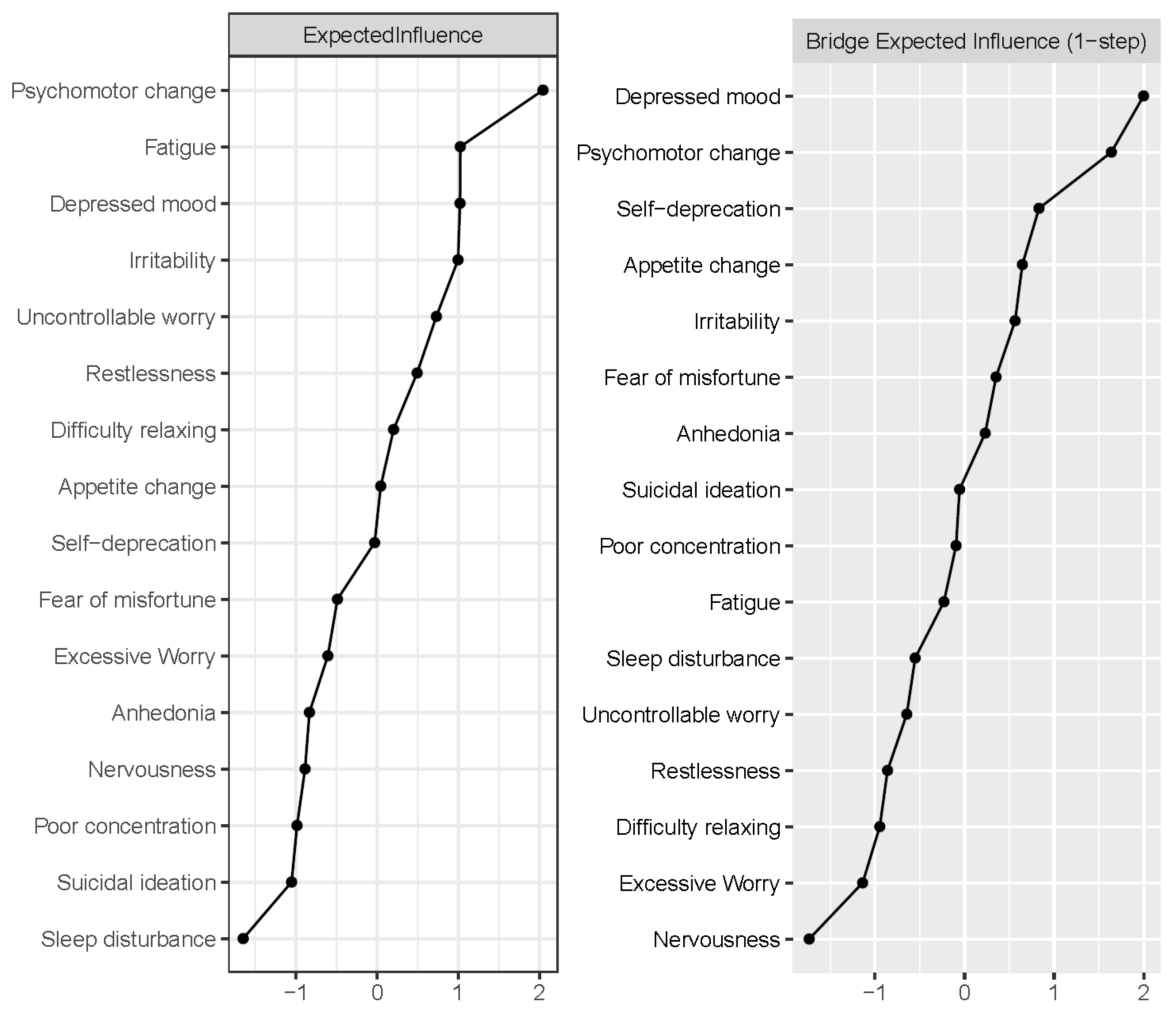
The centrality indices of expected influence (EI) and bridge EI (bEI).

Figure 5 presents the centrality indices of expected influence (EI) and bridge expected influence (bEI). Based on the EI indicators of all symptoms derived from the estimated psychometric network, one symptom with the highest centrality was identified, namely PHQ8 (“psychomotor change”, 1.26). This finding indicated that this node played the most critical role in the entire network and exerted the strongest influence on other nodes. Among the depression-related symptoms, PHQ3 (“sleep disturbance”) and PHQ9 (“suicidal ideation”) exhibited the lowest EI values, which suggested that these two nodes had the weakest interconnections with other symptoms in the network. The bootstrapped difference test confirmed that there was a statistically significant difference between “psychomotor change” and 68.75% of all nodes in the entire network (see Supplementary Materials Figure S2). Furthermore, the order of EI indicators in the network remained unchanged after the removal of partial samples or nodes (CS-coefficient = 0.60, see Figure 3). With regard to the bEI indicators of all symptoms in the network, two nodes with the highest bEI values were identified: PHQ2 (“depressed mood”, 0.70) and PHQ8 (“psychomotor change”, 0.62). This result demonstrated that these two symptoms exhibited the strongest cross-community associations within the network. The CS-coefficient (0.60) and the bootstrapped difference test (with nodes showing significant differences accounting for 56.25%) indicated that the evaluation of bEI was relatively stable (see Supplementary Materials Figure S2 and Figure 3).

### 3.3. Gender-Related Network Comparison

The NCT produced mixed findings for gender-related network differences. Globally, network invariance was non-significant (difference value = 0.26, *p* = 0.673), suggesting similar symptom–symptom association structures for male and female PE teachers. Nevertheless, global strength differed significantly (difference value = 0.82 [male = 7.44, female = 8.25], *p* = 0.021). Furthermore, no significant gender differences in edge-weight, EI, and bEI indicators emerged from local level permutation test following FDR correction.

Both male and female networks exhibited acceptable stability. Bootstrap 95% confidence intervals for edge weights indicated minimal estimation bias: original edge estimates closely overlapped with bootstrap means and fell consistently within confidence bands for both gender networks (see Supplementary Materials Figure S3). Case-dropping subset bootstrap analysis further corroborated these findings. The male network demonstrated satisfactory stability, with CS coefficients of 0.52 for edge weights, 0.60 for EI, and 0.52 for bEI. The female network yielded corresponding CS values of 0.44, 0.36, and 0.44, indicating acceptable but comparatively lower stability. Centrality difference tests revealed that core nodes in the male network differed significantly from 81% of all nodes, whereas in the female network this proportion dropped below 19%, suggesting that the identified central symptoms are considerably less stable in the female network (see Supplementary Materials Figure S3).

## 4. Discussion

### 4.1. Network Analysis of GAD and PHQ

As the first study to employ network analysis targeting Chinese PE teachers, this research explored their anxiety and depressive symptom characteristics, overlapping symptoms, and gender effects. Results indicated that specific depression symptoms were pivotal to the overall network: “psychomotor change” was the most central EI and bEI indicator, while “depressed mood” also served as a key bEI indicator, suggesting that targeted intervention on these symptoms may enhance effectiveness. Additionally, the association between “irritability” and “anhedonia” bridged anxiety and depression symptoms, and four overlapping symptoms (“restlessness”, “irritability”, “self-deprecation”, and “psychomotor change”) identified via the CPM algorithm deepened understanding of the comorbidity of anxiety and depression. Finally, gender-related effects were somewhat mixed. While global strength differed significantly by gender, no gender divergence emerged for network invariance, edge-weight configurations, or node centrality metrics. These findings indicated that the anxiety–depression symptom-association structure was largely comparable between male and female PE teachers. Although these results tentatively suggested the possibility of gender-agnostic intervention approaches for this population, such practical implications await future empirical validation.

We established a relatively accurate and reliable network architecture for anxiety and depression symptoms. Within this network, the three strongest edges were identified: the strongest anxiety-symptom edge (GAD1-GAD2), the strongest depression-symptom edge (PHQ8-PHQ9), and the bridging edge connecting the anxiety and depression symptom (GAD6-PHQ1). Among these edges, the GAD1-GAD2 association was corroborated by bivariate correlation analysis: this pair remained strongly correlated regardless of whether other variables were controlled for, confirming the robustness of this edge in the network. This finding enhances our understanding of the structural coherence of anxiety symptoms. Notably, bivariate correlation analysis identified PHQ4-PHQ5 as the strongest pair among depressive symptoms, whereas in the partial correlation network (controlling for all other symptoms), PHQ8-PHQ9 emerged as the strongest edge. Likewise, for cross-symptom connections between anxiety and depression, the bivariate correlation showed the strongest association between GAD3 and PHQ9, which shifted to GAD6-PHQ1 in the partial correlation network. These discrepancies indicate that the bivariate associations of PHQ4-PHQ5 and GAD3-PHQ9 may be confounded by other depressive symptoms rather than representing direct correlations. In contrast, PHQ8 and PHQ9 exhibit a direct association that is not explained by other symptoms, suggesting that “psychomotor change” and “suicidal ideation” occupy core, independent positions within the depressive structure. Psychomotor change is a psychiatry construct covering two mutually exclusive depressive manifestations: observable psychomotor retardation (slowed movement and speech) and psychomotor agitation (involuntary restless and fidgeting; Beard et al., 2016). Critically, this symptom is distinct from the occupational physical fatigue PE teachers experience from prolonged physical activity—it originates from affective dysfunction (APA, 2022; Kroenke et al., 2001), capturing aberrant motor and speech rhythms of PE teachers secondary to depressed mood and emotional dysregulation. The close association between psychomotor change and suicidal ideation further underscores its significance in the network. Addressing suicidal ideation among teachers remains a sensitive, culturally taboo topic (Ruiz-Ordóñez & Sesé, 2024); despite being tasked with supporting students’ mental health, teachers face substantial stressors—including heightened workload and interpersonal strain—that elevate suicide risk (Ruiz-Ordóñez & Sesé, 2024). The finding tentatively suggest that “psychomotor change” may represent a potentially salient indicator for suicide-risk assessment within this population. Nevertheless, few studies have examined this specific symptom pair, limiting current understanding of depressive symptom architecture.

Consistent with Kaiser et al.’s (2021) network framework, this research identified four symptom clusters with four different overlapping symptoms (i.e., “restlessness”, “irritability”, “self-deprecation”, and “psychomotor change”), stressing that Chinese PE teachers’ psychological symptoms are comorbid clusters centered on these overlaps. Based on Kaiser et al. (2021), overlapping symptoms serve as trans-diagnostic hubs that connect distinct symptom clusters, breaking the traditional categorical boundaries between anxiety and depression (Goldberg, 2000; Vink et al., 2008; Zbozinek et al., 2012) and playing a pivotal role in explaining comorbidity of anxiety and depression between anxiety and depression symptoms. It should be noted that several items from PHQ-9 and GAD-7 show phenomenological content overlap (for example: GAD5 “restlessness” with PHQ8 “psychomotor change”, GAD4 “difficulty relaxing” with PHQ7 “poor concentration”, and PHQ4 “fatigue” with PHQ8 “psychomotor change”). Some symptoms were assigned to multiple communities in CPM outputs. Although these overlapping symptoms reflect well-recognized transdiagnostic clinical presentations, we cannot fully rule out minor influences from shared item descriptions across scales when interpreting overlapping community membership.

Additionally, contrary to the assumption, the study found “psychomotor disturbance” was both the most central (highest EI) and bridge (highest bEI) symptom—scarcely identified as such in prior studies (Beard et al., 2016; Bian et al., 2024; Cai et al., 2024; Luo et al., 2024; Y. Zhang et al., 2024), exerting a critical effect on activating, maintaining, and bridging the underlying psychopathological network. Overall, the dual status of psychomotor change—as both a central node (highest EI and bEI) and an overlapping symptom—underscores its structural significance within the anxiety–depression network of PE teachers. Two plausible, albeit tentative, mechanisms may help explain this unique prominence. First, psychomotor symptoms may carry amplified impact in this population due to the nature of PE teachers’ professional identity, which centers on sustained physical vitality and movement-based instruction. The emergence of psychomotor retardation or agitation could thus be perceived as a direct threat to core occupational competence, potentially exacerbating both depressive and anxious symptoms. Second, heightened bodily awareness—an occupational characteristic of PE teachers—may predispose emotional distress to manifest and escalate preferentially through somatic-motor pathways, possibly creating a reciprocal cycle between affective dysregulation and motor rhythm disruption. Clinically, the findings suggest that psychomotor change may serve as both a readily observable screening marker and a high-leverage intervention target for this occupational group; targeting this symptom may yield cascading benefits across both anxiety and depressive domains in the PE teachers’ population.

### 4.2. Gender Effects

The non-significant gender difference in symptom severity, coupled with the largely comparable network structure observed in the present study, may tentatively suggest that the unique occupational context of Chinese PE teachers could attenuate commonly observed gender-related disparities in mental health. Unlike the general teaching population, where female teachers often exhibit higher symptom loads (Bogaert et al., 2014; Lu, 2018), male and female PE teachers in this sample face nearly identical, gender-transcendent stressors, including prolonged high-intensity physical exertion, marginalized institutional status, and cumulative occupational fatigue (L. He et al., 2025; X. Li et al., 2025; Lu & Zhang, 2018; Zhu & Li, 2019). This shared occupational burden may contribute to the similarity in both overall anxiety–depression severity and underlying symptom–symptom association across genders.

Notably, Network Comparison Tests yielded nuanced, mixed findings that should be interpreted following a clear hierarchical framework: network invariance, global strength, individual edge-weight, and node-level centrality. This global-to-local interpretive sequence represents a widely accepted reporting convention within the psychological network literature, grounded in the methodological properties of each permutation-based test statistic (van Borkulo et al., 2023). At the highest structural level, the non-significant omnibus network invariance test confirmed that male and female PE teachers exhibited congruent symptom-interconnection patterns. Nevertheless, a statistically significant difference emerged in global network strength, while no meaningful gender disparities were detected in individual edge weights or centrality indicators (EI, bEI) following FDR correction. According to the permutation-based logic of NCT, this pattern indicates that the divergence in global strength most likely arises from small, cumulative differences across multiple edges, rather than from large-magnitude gaps in any single symptom-to-symptom connection or from shifts in the central role of specific nodes. The elevated global interconnectedness observed among female PE teachers may reflect amplified mutual reinforcement of emotional symptoms. Although female PE teachers experience many of the same workplace demands as their male colleagues, they often face additional, gender-specific stressors, including dual burdens stemming from household and care-giving responsibilities, subtle workplace prejudice, and professional title promotion (Lu, 2018). These extra-layered pressures may strengthen reciprocal associations between anxiety-depressive symptoms across the network, while leaving the fundamental architecture of symptom–symptom connections unchanged.

Caution is warranted when interpreting the observed null findings from local comparisons. While bootstrap sampling and CS coefficient analyses partially supported the stability of core network structures, centrality stability metrics indicated relatively low stability in the female network. This limitation likely reflects the demographic structure of the Chinese PE teaching profession, where the total PE teacher workforce is smaller than that of other subjects, and female PE teachers are markedly outnumbered by males, creating an inherent gender imbalance in sampling. Despite targeted oversampling of female participants, the female sample size remained insufficient for robust estimation. If replicated, these exploratory findings would imply that gender exerts a relatively modest influence on the core psychopathological mechanisms in this population, and that “psychomotor change” as a broadly relevant intervention target across both genders. For clinical practice, these cross-sectional, exploratory findings provisionally suggest that gender-universal screening and intervention approaches may warrant further investigation as a strategy for engaging shared symptom pathways among PE teachers. Prospective studies with larger samples are needed to confirm these preliminary patterns.

### 4.3. Implication

This study provides substantial theoretical and practical implications for understanding anxiety–depression comorbidity among Chinese PE teachers. Theoretically, it extends network analysis to this understudied occupational population, identifying “psychomotor change” as the most central, bridging, and overlapping symptom. Specifically, these correlational findings tentatively suggest “psychomotor change” may function as a key transdiagnostic hub relevant to suicidality, rather than merely a secondary somatic symptom. The identification of four overlapping symptoms further validates Kaiser et al.’s (2021) comorbidity framework, highlighting shared pathological pathways that transcend traditional anxiety–depression boundaries. Additionally, although global strength differed significantly between groups, symptom interconnections patterns were largely comparable. This pattern offers tentative insights that shared occupational stressors—including high-intensity physical exertion and institutional marginalization—may contribute to similar symptom interplay across male and female PE teachers. Practically, these findings prioritize “psychomotor change” as a tangible, observable candidate intervention target. Given its central role across multiple network functions, interventions targeting “psychomotor change” warrant further evaluation as a potential strategy to reduce comorbidity and suicide risk in this populations. The largely consistent symptom interplay across genders further suggests that efficient, gender-universal screening and intervention strategies for PE teachers, which may optimize resource allocation while addressing the unique mental health challenges of this high-stress occupational population.

### 4.4. Limitations and Future Directions

Several limitations should be acknowledged. First, this study adopted a cross-sectional design, which precludes causal inferences and prevents examination of dynamic changes in symptom networks over time, especially for the mechanism between “psychomotor change” and “suicidal ideation”. Prospective studies using time-series network analysis should track PE teachers across academic semesters or years to identify how overlapping and bridging symptoms drive transitions from anxiety to depression and suicidality, enabling causal testing. Furthermore, randomized controlled trials should evaluate whether targeting “psychomotor change” reduces anxiety/depression severity and suicide risk in PE teachers, comparing outcomes to mood-focused interventions.

Second, the sample was restricted to Chinese PE teachers, limiting generalizability to other regions or occupational groups. Future research should include multi-site samples to enhance external validity. Moreover, while FDR correction controlled for type I error, small and imbalanced sample sizes in gender-stratified subgroups might have reduced power to detect subtle local differences. Future research with larger, demographically balanced samples could strengthen statistical power to identify potential gender-specific local variations in edge strength or centrality, and further clarify whether truly negligible gender differences exist in the symptom network of PE teachers.

Third, occupational physical fatigue may create inherent measurement confounding for PE teachers’ self-reported PHQ4 “fatigue” and PHQ8 “psychomotor change”. As frontline educators engaged in daily high-intensity physical instruction, participants’ subjective reports of slowed movement or tiredness may conflate depressive psychomotor dysfunction with work-induced muscular exhaustion. For future depression assessments targeting PE teachers, preliminary practical evaluation and targeted revision of measurement scales may be implemented in advance to accurately capture depressive symptoms specific to this occupational group; additionally, incorporating objective motor assessments—such as actigraphy or speech rhythm analysis—are warranted to validate these network-derived hypotheses and disentangle psychomotor symptoms from occupational physical fatigue in PE teachers.

Fourth, one notable limitation concerns PHQ-9 Item 8 “psychomotor change”, a double-barreled item covering both psychomotor agitation and psychomotor retardation. We incorporated this composite item as one single node in our network model. As documented in previous psychometric research, bifurcated items may produce ambiguous centrality estimates and conceal differential symptom associations between the two psychomotor phenotypes (Hallquist et al., 2021; Menold, 2020). While separate measurement of agitation and retardation would enhance result precision, we lack disaggregated data to separate these two constructs in the current sample. We therefore caution against overinterpreting the associations of this node, and recommend that follow-up studies adopt differentiated items for these opposing psychomotor symptoms.

Finally, occupation-specific occupational risk factors (e.g., occupational stress, burnout, workload, institutional support) were not assessed in this cross-sectional study. Therefore, we cannot determine to what extent the observed anxiety-depressive symptom network is shaped by the unique occupational characteristics of physical education teachers versus general individual psychological traits. This restricts the formulation of targeted school-based intervention strategies tailored for PE teachers. Future longitudinal research incorporating comprehensive occupational risk indicators is warranted. By integrating machine learning pipelines with longitudinal network modeling, such follow-up work can rigorously identify key occupational risk drivers and unpack tentative directional, causal-like interplay between occupational exposures and evolving affective symptom networks.

## 5. Conclusions

This study represents the first network analysis of anxiety and depression among Chinese PE teachers, yielding several key findings. “Psychomotor change” emerged as the most central symptom, a key bridge symptom, and a critical overlapping symptom simultaneously, underscoring its unique significance among Chinese PE teachers. CPM community detection revealed overlapping symptoms including “restlessness”, “irritability”, “self-deprecation”, and “psychomotor change” as transdiagnostic hubs associated with anxiety–depression comorbidity. Moreover, NCT revealed that symptom interconnections patterns were largely comparable, while global strength differed significantly between male and female networks. This pattern tentatively implies that shared occupational stressors may outweigh commonly observed gender disparities in mental health. Collectively, these cross-sectional gender-comparative results highlight “psychomotor change” as a potential candidate target for future longitudinal or experimental research exploring intervention for comorbid affective symptoms and suicidal risk among PE teachers experiencing occupational stress.

## Figures and Tables

**Table 1:** Descriptive statistics of the GAD-7 and PHQ-9 items.

Items	*N*	*M*	*SD*	K-S Test	Predictivity
GAD1	519	0.72	0.80	0.27 ***	0.57
GAD2	519	0.59	0.81	0.34 ***	0.62
GAD3	519	0.68	0.80	0.30 ***	0.59
GAD4	519	0.65	0.83	0.33 ***	0.64
GAD5	519	0.51	0.79	0.38 ***	0.65
GAD6	519	0.58	0.79	0.35 ***	0.68
GAD7	519	0.45	0.77	0.41 ***	0.53
PHQ1	519	0.58	0.76	0.34 ***	0.58
PHQ2	519	0.50	0.75	0.38 ***	0.64
PHQ3	519	0.65	0.83	0.32 ***	0.48
PHQ4	519	0.63	0.77	0.30 ***	0.65
PHQ5	519	0.52	0.72	0.36 ***	0.59
PHQ6	519	0.42	0.69	0.41 ***	0.58
PHQ7	519	0.48	0.74	0.39 ***	0.53
PHQ8	519	0.41	0.71	0.42 ***	0.69
PHQ9	519	0.26	0.65	0.49 ***	0.45

K-S Test = Kolmogorov–Smirnov Test; *** *p* < 0.001.

**Table 2:** Comparison of anxiety and depression symptoms between genders.

	Male	Female	*χ*^2^/*t*	*p*-Value	Effect Size
GAD					
GAD ≥ 10	49	19	0.23	0.631	0.02
GAD < 10	312	139			
*M* ^1^ (*SD*)	13.73 (2.68)	12.47 (2.50)	10.78	0.081	0.48
*M* ^2^ (*SD*)	4.16 (4.76)	4.25 (4.18)	−0.22	0.823	−0.02
PHQ					
PHQ ≥ 7	101	55	20.44	0.118	0.07
PHQ < 7	260	103			
*M* ^1^ (*SD*)	11.30 (4.34)	10.56 (3.77)	10.06	0.293	0.18
*M* ^2^ (*SD*)	4.29 (5.20)	4.81 (5.03)	−10.07	0.286	−0.10

Effect sizes are reported as Cohen’s *d* (continuous variables) and Cramer’s *V* (categorical variables). *M* ^1^ (*SD*) = mean values (standard deviations) for the subgroup scoring above or equal to the cutoff; *M* ^2^ (*SD*) = total-scale mean values (standard deviations) for the full male and female sample.

## Data Availability

The data supporting the findings of this study are available from the corresponding author upon reasonable request.

## Supplementary Materials

The following supporting information can be downloaded at: https://www.mdpi.com/article/10.3390/bs16091534/s1, Figure S1: Bootstrap CI for the edges of the network; Figure S2: Difference test for the edge, EI and bEI indicators; Figure S3: Stability analysis for male (left) and female (right) networks; Table S1: Sensitivity analysis for the community structure.

## References

[B1-behavsci-16-01534] Agyapong B., Obuobi-Donkor G., Burback L., Wei Y. (2022). Stress, burnout, anxiety and depression among teachers: A scoping review. International Journal of Environmental Research and Public Health.

[B2-behavsci-16-01534] American Psychiatric Association, DSM-5 Task Force (2022). Diagnostic and statistical manual of mental disorders: DSM-5^TR^.

[B3-behavsci-16-01534] Amirhosseini M., Ghaleh O. S., Heydari F., Hami M., Shahriari B. (2021). Influence of physical fitness on job burnout and mental health among female physical education teachers. Journal of Hunan University Natural Science.

[B4-behavsci-16-01534] Basiri R., Seidu B., Rudich M. (2023). Exploring the interrelationships between diabetes, nutrition, anxiety, and depression: Implications for treatment and prevention strategies. Nutrients.

[B5-behavsci-16-01534] Beard C., Millner A. J., Forgeard M. J. C., Fried E. I., Hsu K. J., Treadway M. T., Leonard C. V., Kertz S. J., Björgvinsson T. (2016). Network analysis of depression and anxiety symptom relationships in a psychiatric sample. Psychological Medicine.

[B6-behavsci-16-01534] Bian Z., Xu R., Shang B., Lv F., Sun W., Li Q., Gong Y., Luo C. (2024). Associations between anxiety, depression, and personal mastery in community-dwelling older adults: A network-based analysis. BMC Psychiatry.

[B7-behavsci-16-01534] Bogaert I., De Martelaer K., Deforche B., Clarys P., Zinzen E. (2014). Associations between different types of physical activity and teachers’ perceived mental, physical, and work-related health. BMC Public Health.

[B8-behavsci-16-01534] Boschloo L. (2018). Zooming in and zooming out: A network perspective on the comorbidity of depression and anxiety. Journal of the American Academy of Child & Adolescent Psychiatry.

[B9-behavsci-16-01534] Bringmann L. F., Lemmens L. H. J. M., Huibers M. J. H., Borsboom D., Tuerlinckx F. (2015). Revealing the dynamic network structure of the Beck Depression Inventory-II. Psychological Medicine.

[B10-behavsci-16-01534] Cai H., Chen M., Li X., Zhang L., Su Z., Cheung T., Tang Y., Malgaroli M., Jackson T., Zhang Q., Xiang Y. (2024). A network model of depressive and anxiety symptoms: A statistical evaluation. Molecular Psychiatry.

[B11-behavsci-16-01534] Carroll A., Forrest K., Sanders-O’Connor E., Flynn L., Bower J. M., Fynes-Clinton S., York A., Ziaei M. (2022). Teacher stress and burnout in Australia: Examining the role of intrapersonal and environmental factors. Social Psychology of Education.

[B12-behavsci-16-01534] Choi K. W., Kim Y. K., Jeon H. J. (2020). Comorbid anxiety and depression: Clinical and conceptual consideration and transdiagnostic treatment. Advances in Experimental Medicine and Biology.

[B13-behavsci-16-01534] de Wit L. M., van Straten A., Lamers F., Cuijpers P., Penninx B. W. (2015). Depressive and anxiety disorders: Associated with losing or gaining weight over 2 years?. Psychiatry Research.

[B14-behavsci-16-01534] Epskamp S., Borsboom D., Fried E. I. (2018). Estimating psychological networks and their accuracy: A tutorial paper. Behavior Research Methods.

[B15-behavsci-16-01534] Epskamp S., Fried E. I. (2018). A tutorial on regularized partial correlation networks. Psychological Methods.

[B16-behavsci-16-01534] Fried E. I., Nesse R. M. (2015). Depression sum-scores don’t add up: Why analyzing specific depression symptoms is essential. BMC Medicine.

[B17-behavsci-16-01534] Gaspersz R., Nawijn L., Lamers F., Penninx B. W. J. H. (2018). Patients with anxious depression: Overview of prevalence, pathophysiology and impact on course and treatment outcome. Current Opinion in Psychiatry.

[B18-behavsci-16-01534] Goldberg D. (2000). Plato versus Aristotle: Categorical and dimensional models for common mental disorders. Comprehensive Psychiatry.

[B19-behavsci-16-01534] Hallquist M. N., Wright A. G. C., Molenaar P. C. M. (2021). Problems with centrality measures in psychopathology symptom networks: Why network psychometrics cannot escape psychometric theory. Multivariate Behavioral Research.

[B20-behavsci-16-01534] Harlev D., Vituri A., Shahar M., Wolpe N. (2025). Depression and anxiety symptom networks across the lifespan. Age and Ageing.

[B21-behavsci-16-01534] He C. (2025). Merely prasing is far from enough.

[B22-behavsci-16-01534] He L., Huang L., Huang Y., Li H., Zhang Z., Li J., Lin S., Wu K., Huang D., Wu F. (2025). Prevalence and influencing factors of anxiety, depression, and burnout among teachers in China: A cross-sectional study. Frontiers in Psychiatry.

[B23-behavsci-16-01534] Jin J., Yu G. (2025). Teachers’ mental health: The current situation and risk prevention. Education Research.

[B24-behavsci-16-01534] Kaiser T., Herzog P., Voderholzer U., Brakemeier E. L. (2021). Unraveling the comorbidity of depression and anxiety in a large inpatient sample: Network analysis to examine bridge symptoms. Depression and Anxiety.

[B25-behavsci-16-01534] Kayaalp A., Page K. J., Rospenda K. M. (2020). Caregiver burden, work-family conflict, family-work conflict, and mental health of caregivers: A mediational longitudinal study. Work and Stress.

[B26-behavsci-16-01534] Kroenke K., Spitzer R. L., Williams J. B. W. (2001). The PHQ-9: Validity of a brief depression severity measure. Journal of General Internal Medicine.

[B27-behavsci-16-01534] Lange J. (2022). An introduction to the clique percolation community detection algorithm *[R package vignette]*.

[B28-behavsci-16-01534] Lemoyne J., Laurencelle L., Lirette M., Trudeau F. (2007). Occupational health problems and injuries among Quebec’s physical educators. Applied Ergonomics.

[B29-behavsci-16-01534] Leng J., Kong X. (2014). Investigation and analysis of psychological health of physical education teachers in common university in Wuhan city. Hubei Sports Science.

[B30-behavsci-16-01534] Li J., Jin Y., Xu S., Luo X., Wilson A., Li H., Wang X., Sun X., Wang Y. (2023). Anxiety and depression symptoms among youth survivors of childhood sexual abuse: A network analysis. BMC Psychology.

[B31-behavsci-16-01534] Li J., Luo C., Liu L., Huang A., Ma Z., Chen Y., Deng Y., Zhao J. (2024a). Depression, anxiety, and insomnia symptoms among Chinese college students: A network analysis across pandemic stages. Journal of Affective Disorders.

[B32-behavsci-16-01534] Li J., Wang L., Pan L., Hu Z., Yin R., Liu J. F. (2024b). Exercise motivation, physical exercise, and mental health among college students: Examining the predictive power of five different types of exercise motivation. Frontiers in Psychology.

[B33-behavsci-16-01534] Li X., Xu H., Zhang J., Cao X. (2025). The relationship between occupational stress and burnout among primary and secondary school physical education teachers: Evidence from multiverse-style analysis and diary method. BMC Psychology.

[B34-behavsci-16-01534] Liu A., Liu X. (2015). The Relationship between Personality Characteristics and Mental Health of Primary and Secondary School Teachers in Jiangsu Province. Journal of Inner Mongolia Normal University (Educational Science).

[B35-behavsci-16-01534] Liu Q., Cai X., Qian X. (2006). Personality characteristics and mental health level of college physical education teachers. Chinese Journal of Clinical Rehabilitation.

[B36-behavsci-16-01534] Lu J. (2018). An analysis of the mental health status of female physical education teachers in institutions of higher learning from a gender perspective. Modern Vocational Education.

[B37-behavsci-16-01534] Lu J., Zhang F. (2018). Brief discussion on the improvement of mental health for sport teachers. Bulletin of Sport Science & Technology.

[B38-behavsci-16-01534] Luo J., Bei D. L., Zheng C., Jin J., Yao C., Zhao J., Gong J. (2024). The comorbid network characteristics of anxiety and depressive symptoms among Chinese college freshmen. BMC Psychiatry.

[B39-behavsci-16-01534] Ma L., Chee C. S., Amri S., Gao X., Wang Q., Wang N., Liu P. (2025). Impact of self-efficacy and burnout on professional development of physical education teachers in the digital age: A systematic review. PeerJ.

[B40-behavsci-16-01534] Melguizo-Ibáñez E., González-Valero G., Badicu G., Yagin F. H., Alonso-Vargas J. M., Ardigò L. P., Puertas-Molero P. (2025). Relationship between motivational climate, anxiety and average mark in pre-service physical education teachers: A cross-sectional study based on structural equation modelling approach. BMC Medical Education.

[B41-behavsci-16-01534] Menold N. (2020). Double barreled questions: An analysis of the similarity of elements and effects on measurement quality. Journal of Official Statistics.

[B42-behavsci-16-01534] Morneau-Vaillancourt G., Coleman J. R. I., Purves K. L., Cheesman R., Rayner C., Breen G., Eley T. C. (2020). The genetic and environmental hierarchical structure of anxiety and depression in the UK Biobank. Depression and Anxiety.

[B43-behavsci-16-01534] Ou L., Shen Q., Xiao M., Wang W., He T., Wang B. (2025). Prevalence of co-morbid anxiety and depression in pregnancy and postpartum: A systematic review and meta-analysis. Psychological Medicine.

[B44-behavsci-16-01534] Palla G., Derényi I., Farkas I., Vicsek T. (2005). Uncovering the overlapping community structure of complex networks in nature and society. Nature.

[B45-behavsci-16-01534] Park S.-C., Kim D. (2020). The centrality of depression and anxiety symptoms in major depressive disorder determined using a network analysis. Journal of Affective Disorders.

[B46-behavsci-16-01534] Peng P., Chen Q., Liang M., Liu Y., Chen S., Wang Y., Yang Q., Wang X., Li M., Wang Y., Hao Y., He L., Wang Q., Zhang J., Ma Y., He H., Zhou Y., Li Z., Xu H., Liu T. (2022). A network analysis of anxiety and depression symptoms among Chinese nurses in the late stage of the COVID-19 pandemic. Frontiers in Public Health.

[B47-behavsci-16-01534] Reichardt J., Bornholdt S. (2006). Statistical mechanics of community detection. Physical Review E.

[B48-behavsci-16-01534] Ren L., Wang Y., Wu L., Wei Z., Cui L.-B., Wei X., Hu X., Peng J., Jin Y., Li F., Yang Q., Liu X. (2021). Network structure of depression and anxiety symptoms in Chinese female nursing students. BMC Psychiatry.

[B49-behavsci-16-01534] Robinaugh D. J., Millner A. J., McNally R. J. (2016). Identifying highly influential nodes in the complicated grief network. Journal of Abnormal Psychology.

[B50-behavsci-16-01534] Ruiz-Ordóñez Y., Sesé A. (2024). Network based evidence of suicidal ideation among teachers. Scientific Reports.

[B51-behavsci-16-01534] Scherrer J. F., Chrusciel T., Zeringue A., Garfield L. D., Hauptman P. J., Lustman P. J., Freedland K. E., Carney R. M., Bucholz K. K., Owen R., True W. R. (2010). Anxiety disorders increase risk for incident myocardial infarction in depressed and nondepressed Veterans Administration patients. American Heart Journal.

[B52-behavsci-16-01534] Shen X., Wang J. (2023). More than the aggregation of its components: Unveiling the associations between anxiety, depression, and suicidal behavior in adolescents from a network perspective. Journal of Affective Disorders.

[B53-behavsci-16-01534] Spitzer R. L., Kroenke K., Williams J. B. W., Löwe B. (2006). A brief measure for assessing generalized anxiety disorder: The GAD-7. Archives of Internal Medicine.

[B54-behavsci-16-01534] Su W., Liu Q. (2025). The impact of physical education teacher support on sport participation among college students: The chain mediating effects of physical education learning motivation and self-efficacy. Frontiers in Psychology.

[B55-behavsci-16-01534] Szuhany K. L., Simon N. M. (2022). Anxiety disorders: A review. JAMA.

[B56-behavsci-16-01534] Tiller J. W. (2012). Depression and anxiety. Medical Journal of Australia.

[B57-behavsci-16-01534] van Borkulo C. D., van Bork R., Boschloo L., Kossakowski J. J., Tio P., Schoevers R. A., Borsboom D., Waldorp L. J. (2023). Comparing network structures on three aspects: A permutation test. Psychological Methods.

[B58-behavsci-16-01534] Vink D., Aartsen M. J., Schoevers R. A. (2008). Risk factors for anxiety and depression in the elderly: A review. Journal of Affective Disorders.

[B59-behavsci-16-01534] von Haaren-Mack B., Schaefer A., Pels F., Kleinert J. (2020). Stress in physical education teachers: A systematic review of sources, consequences, and moderators of stress. Research Quarterly for Exercise and Sport.

[B60-behavsci-16-01534] Wang W., Bian Q., Zhao Y., Li X., Wang W., Du J., Zhang G., Zhou Q., Zhao M. (2014). Reliability and validity of the Chinese version of the Patient Health Questionnaire (PHQ-9) in the general population. General Hospital Psychiatry.

[B61-behavsci-16-01534] Wang X. Y., Wang Z. W., Jiang D. L., Liu C., Xing W. Y., Yuan Z. T., Cui L.-B., Wu S.-J., Ren L. (2025). Personality perspective on depression and anxiety symptoms among Chinese adolescents and young adults: A two-sample network analysis. BMC Psychiatry.

[B62-behavsci-16-01534] Wu C., Zhou Z., Ni L., Cao J., Tan M., Wu X., Xu Y., Hu J. (2021). Correlation between anxiety-depression symptoms and immune characteristics in inpatients with 2019 novel coronavirus in Wuhan, China. Journal of Psychiatric Research.

[B63-behavsci-16-01534] Xu L. Y. (2019). An attribution analysis of work frustration among young physical education teachers and its impact on their careers. Journal of Tongling Vocational & Technical College.

[B64-behavsci-16-01534] Zbozinek T. D., Rose R. D., Wolitzky-Taylor K. B., Sherbourne C., Sullivan G., Stein M. B., Roy-Byrne P. P., Craske M. G. (2012). Diagnostic overlap of generalized anxiety disorder and major depressive disorder in a primary care sample. Depression and Anxiety.

[B65-behavsci-16-01534] Zhang P., Wang L., Zhou Q., Dong X., Guo Y., Wang P., He W., Wang R., Wu T., Yao Z., Hu B., Wang Y., Zhang Q., Sun C. (2023). A network analysis of anxiety and depression symptoms in Chinese disabled elderly. Journal of Affective Disorders.

[B66-behavsci-16-01534] Zhang Y., Cui Y., Li Y., Lu H., Huang H., Sui J., Guo Z., Miao D. (2024). Network analysis of depressive and anxiety symptoms in older Chinese adults with diabetes mellitus. Frontiers in Psychiatry.

[B67-behavsci-16-01534] Zhu D., Li A. (2019). Causes, influences and countermeasures of physical education teachers’ stigma. Hubei Sports Science.

[B68-behavsci-16-01534] Zuo H. (2009). Investigation on the current mental health status of middle school physical education teachers. Neijiang Technology.

