# Supplementary material for: Psychometric Network Characteristics of Anxiety and Depression Among Chinese Physical Education Teachers
Source: Behav Sci (Basel). 2026 Aug 31;16(9):1534. doi: 10.3390/bs16091534 (PMC13603403; doi:10.3390/bs16091534)
Supplement: Supplementary file 1 [file behavsci-16-01534-s001.zip › behavsci-4432591-supplementary.pdf]

Supplementary Materials

Figure S1: Bootstrap CI for the edges of the network

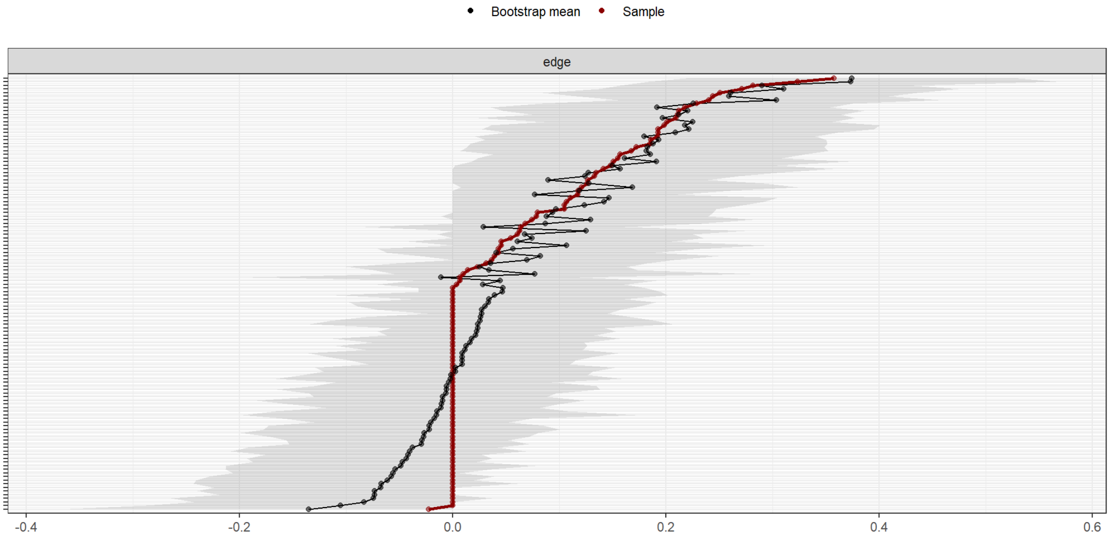

Figure S2: Difference test for the edge, EI and bEI indicators

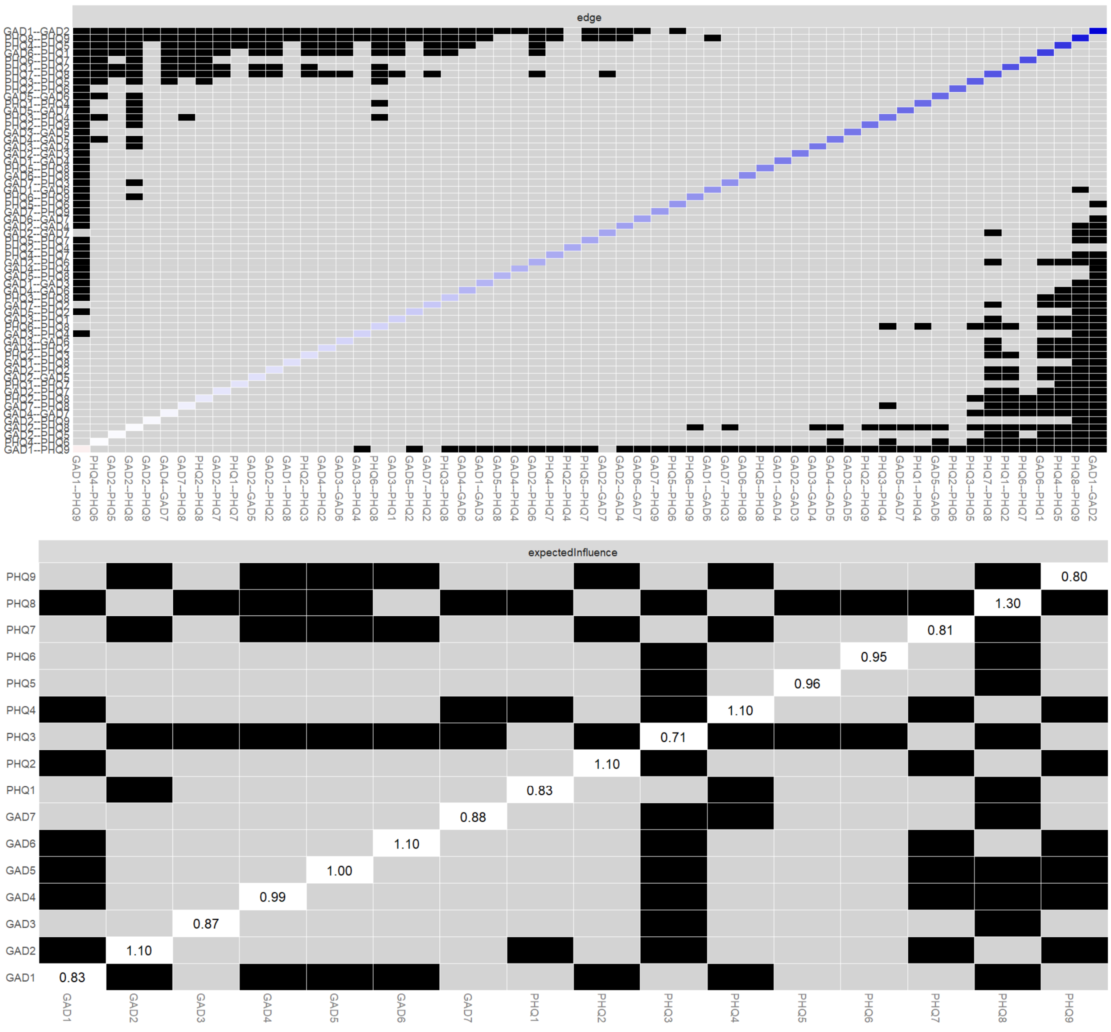

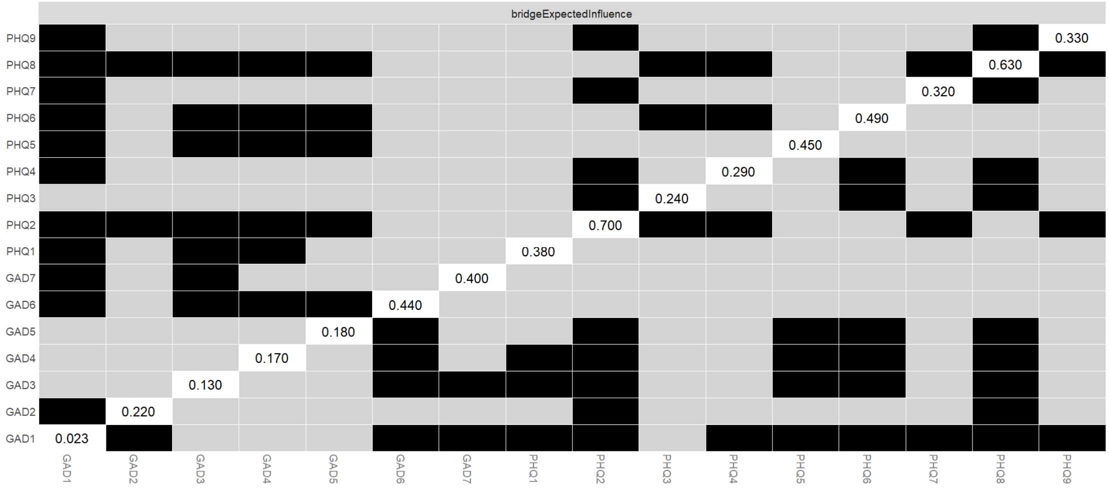

Figure S3: Stability analysis for male (left) and female (right) networks

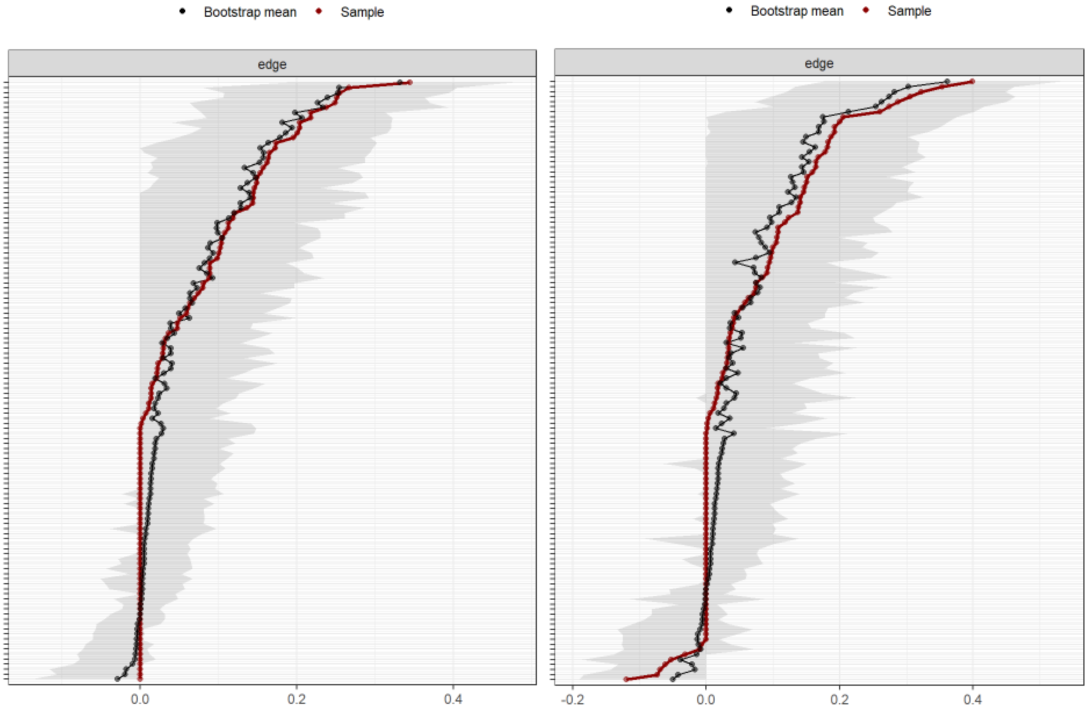

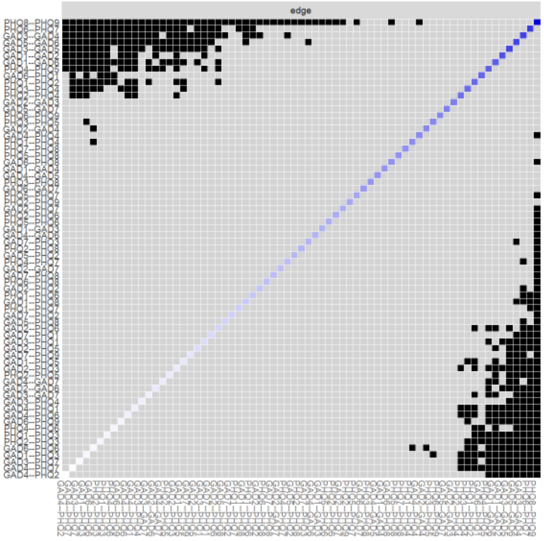
edge

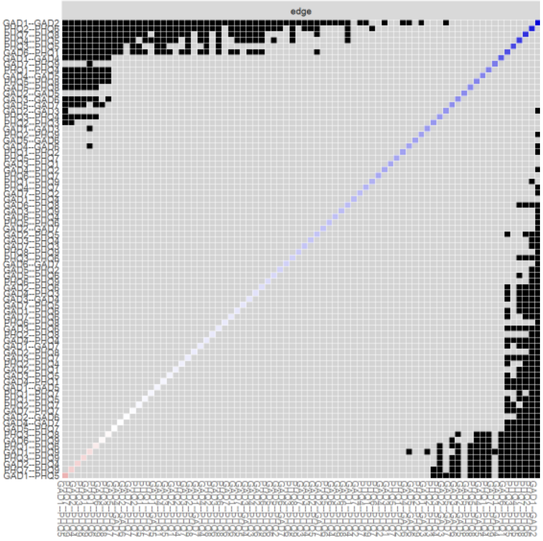
edge

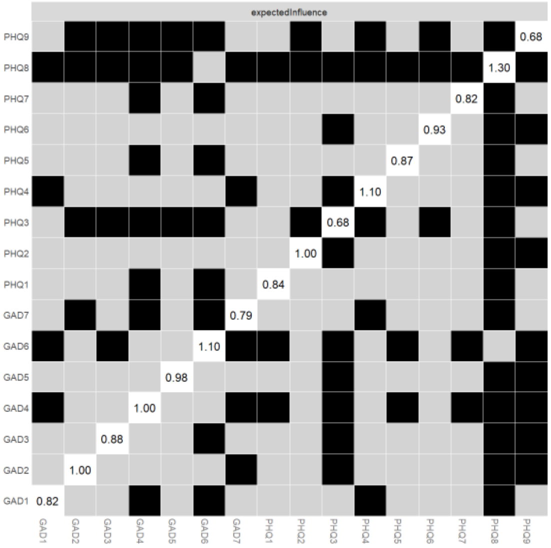
expectedInfluence
GAD1 GAD2 GAD3 GAD4 GAD5 GAD6 GAD7 PHQ1 PHQ2 PHQ3 PHQ4 PHQ5 PHQ6 PHQ7 PHQ8 PHQ9
0.82
1.00
0.88
1.00
0.98
1.10
0.79
0.84
1.00
0.68
1.10
0.87
0.93
0.82
1.30
0.68

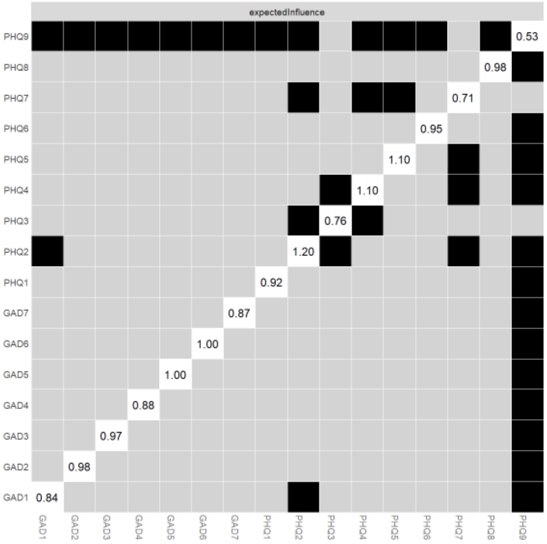
expectedInfluence
GAD1 GAD2 GAD3 GAD4 GAD5 GAD6 GAD7 PHQ1 PHQ2 PHQ3 PHQ4 PHQ5 PHQ6 PHQ7 PHQ8 PHQ9
0.84
0.98
0.97
0.88
1.00
1.00
0.87
0.92
1.20
0.76
1.10
1.10
0.95
0.71
0.98
0.53

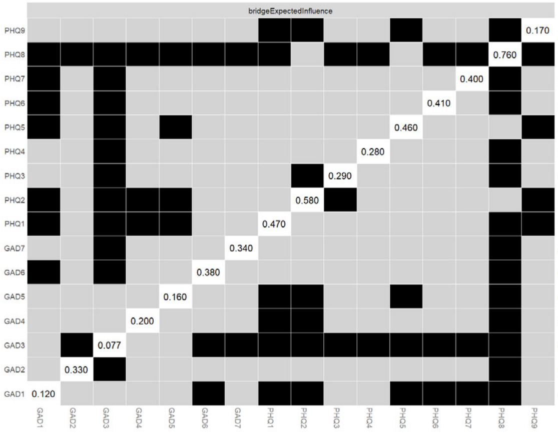
bridgeExpectedInfluence
GAD1 GAD2 GAD3 GAD4 GAD5 GAD6 GAD7 PHQ1 PHQ2 PHQ3 PHQ4 PHQ5 PHQ6 PHQ7 PHQ8 PHQ9
0.120
0.330
0.077
0.200
0.160
0.380
0.340
0.470
0.580
0.290
0.280
0.460
0.410
0.400
0.760
0.170

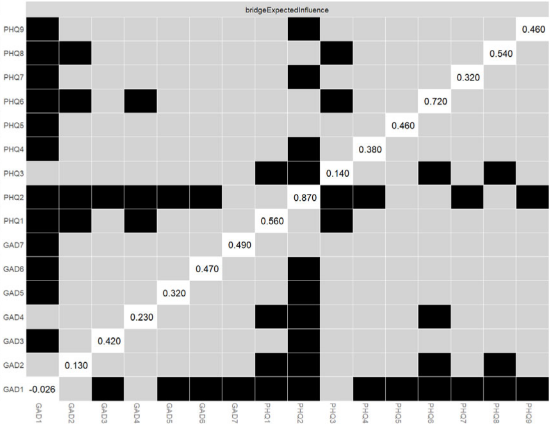
bridgeExpectedInfluence
GAD1 GAD2 GAD3 GAD4 GAD5 GAD6 GAD7 PHQ1 PHQ2 PHQ3 PHQ4 PHQ5 PHQ6 PHQ7 PHQ8 PHQ9
-0.026
0.130
0.420
0.230
0.320
0.470
0.490
0.560
0.870
0.140
0.380
0.460
0.720
0.320
0.540
0.460

Table S1: Sensitivity analysis for the community structure

We conducted a sensitivity analysis excluding four overlapping items to test whether the identified community structure remained stable and to assess whether the observed clustering might be an artifact driven by shared content across scales. The results are reported below.

Community		1	2	3	4
Full scale	memberships	GAD1<br>GAD2<br>GAD3<br>GAD4<br>**GAD5**<br>**GAD6**	PHQ2<br>**PHQ6**<br>**PHQ8**<br>PHQ9	PHQ5<br>**PHQ6**<br>PHQ7<br>**PHQ8**	**GAD5**<br>**GAD6**<br>GAD7<br>**PHQ8**
	Shared nodes	GAD5 GAD6 PHQ6 PHQ8			
	Isolated nodes	PHQ1 PHQ3 PHQ4			
Incomplete scale	memberships	GAD1<br>GAD2<br>GAD3<br>GAD4	PHQ1<br>PHQ2<br>PHQ4	PHQ3<br>PHQ4<br>PHQ5<br>PHQ7	GAD7<br>PHQ2<br>PHQ9
	Shared nodes	PHQ2 PHQ4			
	Isolated nodes	NULL			

Full scale = full 16 items with 7 items from GAD and 9 items from PHQ. Incomplete scale = 12 items including 4 overlapping items detected in this study.

It is noteworthy that even after removing the four putatively overlapping items, new shared nodes (PHQ2, PHQ4) still emerged in the CPM output. This algorithmic behavior is intrinsic to the clique-percolation method: shared nodes are produced when nodes participate in multiple distinct k-cliques within the partial-correlation network, rather than purely driven by lexical similarity of item wording. Node removal reshapes the global network topology and reconfigures existing k-clique structures, which may shift overlapping membership onto other remaining symptoms. This observation further supports our argument that cross-community overlap reflects inherent transdiagnostic connectivity of anxiety-depression symptom networks, and is not merely an artifact caused by these four candidate overlapping items. Although isolated nodes disappeared in the reduced-item analysis, the overall partitioning into four major communities remained largely consistent.
